# Role of ICAN in rate, spike time, and theta phase coding by persistent spiking neurons of the medial entorhinal cortex

**DOI:** 10.1186/1471-2202-12-S1-P140

**Published:** 2011-07-18

**Authors:** Nathan W Schultheiss, Erik Fransen, Michael E Hasselmo

**Affiliations:** 1 Department of Psychology, Center for Memory and Brain, Boston University, Boston, Massachusetts 02215, USA; 2 School of Computer Science and Communication, Royal Institute of Technology, Stockholm, Sweden

## 

Persistent spiking neurons of the medial entorhinal cortex (mEC) exhibit bistability in that they are quiescent in the absence of synaptic or applied currents, but can be switched to a stable spiking state by a brief, strong depolarizing input. The spiking state is thought to be maintained by a positive feedback loop between a nonspecific calcium-sensitive cationic current (ICAN) carried by TRPC membrane channels and heightened intracellular calcium levels sustained during sufficiently fast spiking. Persistent spiking neurons in layer V of mEC are capable of sustaining a continuous range of stable spiking frequencies and can be switched to higher or lower frequencies by additional strong depolarizing or hyperpolarizing pulses, respectively**^1^**. This ability to generate *graded* persistent spiking has been hypothesized to underlie aspects of working memory, but it remains unclear if and to what extent persistent spiking is robust to the considerable fluctuating synaptic conductance that would be expected *in vivo*. Furthermore, the contribution of ICAN to the input-output processing of these neurons has not been evaluated. In this study we use a multi-compartmental GENESIS model of a layer V mEC neuron**^2^** to investigate how ICAN might contribute to rate, spike time, and theta phase coding in these neurons. In the absence of synaptic inputs, we vary either an applied holding current or the magnitude of a 1 second current pulse delivered to the base model or to a version of the model from which ICAN has been removed. We then evaluate the spike frequency to applied current relationship at different time-points over the next 10 seconds. To explore the potential role of ICAN in spike time coding we deliver a 500 pA, 1 second current pulse during ongoing stochastic backgrounds composed of 1 nS AMPA and GABA synaptic events delivered to the soma. These backgrounds are intended to represent the *in vivo* high conductance state. We then evaluate the spike-triggered average (STA) of the CAN current before and after the pulse. We also derive the STAs of voltage (mean spike shape) and AMPA and GABA input conductances to evaluate how activation of ICAN might affect spike threshold and sensitivity to synaptic input fluctuations. Lastly, we delivered theta modulated synaptic input to the model and evaluate the preferred firing phase of the model for different levels of ICAN activation. We find that: 1) ICAN shapes the spike frequency to current relationship in a time-dependent manner; 2) The CAN current can support long-lasting elevated firing rates following strong excitation amidst ongoing stochastic synaptic activity; 3) Activation of the CAN current with a current pulse increases sensitivity to future synaptic input fluctuations; 4) Increased sensitivity is long-lasting and is greater for stronger pulses, allowing the CAN current to act as an intrinsic gain control mechanism.
